# Zoledrinic Acid Induces Steoblastic Differentiation of Mesenchymal Stem Cells without Change in Hypomethylation Status of OSTERIX Promoter

**Published:** 2012-08-31

**Authors:** Majid Farshdousti Hagh, Mehrdad Noruzinia, Yousef Mortazavi, Masoud Soleimani, Saeid Kaviani, Maryam Mahmodinia Maymand

**Affiliations:** 1. Department of Hematology, Tarbiat Modares University, Tehran, Iran; 2. Department of Hematology, Zanjan University of Medical Science, Zanjan, Iran; 3. Sarem Cell Research Center- SCRC, Sarem Women’s Hospital, Tehran, Iran

**Keywords:** Zoledronic Acid, Osteoblastic Differentiation, Mesenchymal Stem Cells, Methylation, OSX

## Abstract

**Objective:**

Mechanism of zoledronic acid on osteoblastic differentiation of mesenchymal stem cells (MSCs) has not fully understood. With the knowledge of some drugs mechanism that alter methylation pattern of some genes, the present research sets out to evaluate osterix (OSX) promoter methylation pattern during zoledronic acid-induced osteoblastic differentiation of MSCs.

**Materials and Methods:**

In this experimental study, MSCs were isolated from human bone marrow. For osteogenic differentiation, MSCs were pulse treated with 5ìM Zoledronic acid for 3 hours and incubated after a medium change in osteogenic differentiation medium for 3 weeks. DNA and RNA were extracted on days 0, 7, 14 and 21 of MSCs differentiating to osteoblast. After cDNA synthesis, OSX expression was evaluated by RT-PCR and quantitative Real-Time PCR. After multiplicity of infection (MOI) treatment, gene specific methylation of OSX was analyzed by methylation specific PCR (MSP).

**Results:**

The mRNA expression of OSX was increased in osteoblast differentiated cells induced by zoledronic acid, especially on days 14 and 21 of differentiation (p<0.05), but expression of OSX didn’t change in undifferentiated MSCs. MSP revealed that, on day 0, undifferentiated MSCs are totally methylated. But, on day 7 of differentiation, MSCs treated by zoledronic acid were totally unmethylated. OSX promoter remained unmethylated, afterwards.

**Conclusion:**

MSP revealed that OSX had a dynamic pattern in methylation, while MSCs gradually differentiated to osteoblasts. Our finding showed that promoter region of OSX is hypomethylated independently from zoledronic acid treatment during osteoblastic differentiation. This knowledge is important to understand drug mechanisms and can be useful for developing new therapies to combat against bone diseases.

## Introduction

Zoledronic acid is a nitrogen-containing bisphosphonate (1). It is used to prevent skeletal fractures in patients with cancers, as well as for treating osteoporosis (2). Zoledronic acid is a potent inhibitor of bone resorption. It inhibits osteoclast proliferation (3) and induces osteoclast apoptotic cell death (4). Its potency results from its high affinity for mineralized bone, especially for sites of high bone turnover (5). Several studies confirm that the zoledronic acid induces osteoblastic differentiation in mesenchymal stem cells (MSCs). Direct treatment of human fetal osteoblasts cells with pamidronate and zoledronic acid was found to decrease cell proliferation, but enhance differentiation (6, 7). Treatment of human osteoblast-like cells derived from trabecular bone explants with zoledronic acid promotes differentiation and mineralization (8). Pulse treatment with zoledronic acid causes sustained commitment of MSCs for osteogenic differentiation (9). The mechanism of osteoblastic differentiation induction by zoledronic acid is still poorly understood. Therefore, study the effects of zoledronic acid on osteoblast differentiation is required.

The process of cell differentiation is fundamental to organ development, maintenance, repair, and regeneration. Multipotential mesenchymal stem cells contribute to the development of several tissues including cartilage and bone. The differentiation of these multipotential cells into discrete lineages, such as chondroblasts; adipoblasts; and osteoblasts, is under strict molecular control. Several factors are involved in the osteoblast differentiation pathway. Osterix is a zinc finger-containing transcription factor expressed in osteoblasts that has important role in osteoblast differentiation pathway (10). Osterix (OSX) belongs to the specificity protein (Sp) subgroup of the Krüppel-like family of transcription factors, that are characterised by a three zinc-finger DNA-binding domain that is located towards the carboxy-terminus of the protein (11-13). Outside the zinc-finger DNA-binding domain, the homology between the Sp proteins is much less and often has no significant homology, at all. The Sp proteins contain central activation domains that are often glutamine-rich or serine/threonine-rich (11). Some genes of Sp proteins contain a TATA box in their promoters that is involved in binding the transcription factor TFIID and the subsequent initiation of transcription. However, other genes of Sp proteins lack of a TATA box, and instead contain another major eukaryotic promoter element, the GC-box with the consensus sequences GGGCGG (14). The RUNX2 (Runt-related transcription factor 2) is regarded as the master transcription factor for osteoblast differentiation since osteoblast differentiation is arrested in RUNX2 null mice (15-17). In these RUNX2 null mice, OSX is not expressed; whereas, in OSX null mice, RUNX2 is expressed, suggesting that RUNX2 acts upstream of OSX (10). OSX regulates the expression of a number of important osteoblast genes, such as osteocalcin, osteonectin, osteopontin, bone sialoprotein and type I collagen (18). However, no information is available on the effect of zoledronic acid in OSX expression in osteoblast lineage commitment.

Epigenetic mechanisms, such as DNA methylation are essential to control the heritable cellular memory of gene expression during differentiation. Recent studies on embryonic and adult stem cells differentiation have highlighted a general and critical role for dynamic epigenetic regulation (19- 22). Our research indicates that receptor tyrosine kinase-like orphan receptor 2 (ROR2) hypomethylated during osteoblastic differentiation (23). Several drugs alter epigenetic pattern. Valproate induces widespread epigenetic reprogramming which involves demethylation of specific genes during treatment of epilepsy (24). 5-aza-2'-deoxycytidine Inhibiting DNA methylation results suppression of the growth of human tumor cell lines and induction differentiation (25).

Altogether, the mechanism of bisphosphonate, such as zoledronic acid action on bone is not fully understood. In this research, we characterize the effect of zoledronic acid on methylation status of OSX promoter region and its expression during osteoblast differentiation.

## Materials and Methods

### Human mesenchymal stem cells preparation

In this experimental study, bone marrow derived human MSCs were obtained from Stemcells Technology Inc. (Tehran, Iran) and maintained according to the manufacturer’s instructions. MSCs were certified for immunophenotyping characterization by flow cytometry and positive differentiation potential for adipogenic, chondrogenic, and osteogenic linages by Stemcells Technology Inc. MSCs were plated in T75 culture flasks and grown in basal media, approximately to 70% confluence. Basal medium for MSCs contained DMEM-low glucose (Gibco, Grand Island, NY, US) was supplemented with 15% (v/v) fetal bovine serum (FBS), 2-mM glutamine, 100µg/ml of Streptomycin, and 100 U/ml of Penicillin. Medium was renewed every two days. Osteogenic differentiation medium was prepared by supplementing the growth medium with 50µg/ml L-ascorbic acid, 10 mM glycerol phosphate, and 100 nM dexamethasone (Sigma, St Louis, MO, USA). Cells cultured and differentiated in T75 culture flasks and 6-well plate. Undifferentiated MSCs and osteoblastic differentiated cells were harvested on 0, 7^th^, 14^th^ and 21^st^ days of differentiation with trypsin-ethylenediaminetetraacetic acid (trypsin-EDTA) solution for DNA and RNA extraction. The 6-well plates were used for Alizarin Red Staining

### MSCs treatment with zoledronic acid

Zoledronic acid (2-(imidazol-1-yl)-hydroxyehtylidene-1,1-bisphosphonic acid, disodium salt) was prepared from Novartis Pharma AG (Basel, Switzerland) and dissolved in double distilled water to make a 5 mM stock solution. For pulse treatment with zoledronic acid, cells were treated in culture medium with final concentration of 5ìM Zoledronic Acid for 3 hours, and incubated after a medium change in osteogenic differentiation medium for 3 weeks (9). Moreover, osteoblastic differentiation of MSCs was carried out as negative control without zoledronic acid treatment as negative control.

### Alizarin red staining

Mineralized extracellular matrix was detected by Alizarin Red Staining (ARS). MSCs were seeded in 6-well plate. Some of wells pulse treated with zoledronic acid and some of wells were without zoledronic acid treatment. Then, cells were cultured in osteogenic differentiation medium for 21 days. For alizarin red staining, cells were fixed with 70% ethanol; rinsed five times with deionized water; treated 10 minutes with 40 mM alizarin red stain at pH=4.2; and washed with phosphate buffered saline (PBS) for 15 minutes with gentle agitation. Cells were examined by an invert microscope.

### DNA extraction and SBS treatment

DNA was extracted from 3-4×10^6^ undifferentiated MSCs and osteoblastic differentiated cells on 7^th^, 14^th^ and 21^st^ days of deifferentiation using DNA Extraction Kit (Roche) according to manufacturer's instructions. DNA purity was assessed with a spectrophotometer and calculated by ratio of the DNA optical density (OD 260) and protein optical density (OD 280). DNA size was analyzed by electrophoresis pattern of sample aliquots (5 µl) in a 1% agarose gel stained with ethidium bromide and visualized under ultraviolet light.

Extracted DNA was subjected to sodium bisulfite treatment to modify unmethylated cytosine to uracil. Sodium bisulfite and hydroquinone solutions were freshly prepared just before their use. Initial denaturation of 1 µg DNA was achieved by NaOH in final concentration of 0.2 M, and the sample was incubated at 37℃ for 20 minutes. The denaturated genomic DNA was treated with sodium bisulfite (pH=5)/hydroquinone to a final concentration of 3.5 M and 10 mM, respectively, in a volume of 500 µl, then incubated at 55℃ for 16 hours under a layer of mineral oil. After this treatment, modified DNA was purified with QIAGEN DNA purification columns according to the manufacturer's instruction and eluted into 200 µl of elution buffer. Then, desulfonation was achieved by addition of NaOH to a final concentration of 0.3 M and incubation at the room temperature for 5 minutes. The solution was neutralized by addition of ammonium acetate (pH=7.0) to a final concentration of 3 M. The DNA was precipitated with adding 4 volume of ethanol, drying, and resuspending in 30 µl double-destilled H_2_O. DNA treated by SBS was either used immediately for methylation specific PCR (MSP) or stored at-20℃.

### Methylation of genomic DNA with SssI methylase

For preparation of methylated DNA as positive control for MSP, extracted DNA of peripheral blood was methylated *in vitro* using Sss1 methylase (Biolabs, New England, US) according to manufacturer's instructions (26). Briefly, the mixture was prepared in a total volume of 20 µl containing 5 µl peripheral blood DNA, 2 µl 10x NE Buffer, 1 µl SssI methylase, 2 µl freshly prepared SAM (S-adenosyl methionine), and 10 µl nuclease-free water. The methylation mixture was incubated at 37℃ for 2 hours followed by 20 minutes at 65℃ to stop the enzyme activity. Finally, DNA was extracted immediately using Roche DNA Extraction Kit and subjected to SBS treatment. Methylated DNA was used as a positive control in MSP, subsequently.

### RNA isolation, cDNA synthesis and RT-PCR analysis

Total RNA was extracted using the RNeasy plus Mini Kit (QIGEN) according to manufacturer’s instructions from undifferentiated MSCs and osteoblast differentiated cells on 7^th^, 14^th^ and 21^st^ days of differentiation. The quality of extracted RNA was determined by gel electrophoresis prior to reverse transcription.

1 µg of total RNA was reversibly transcribed to cDNA using 4µl 5X reaction buffer (containing 25 mM Tris-HCl (pH=8.3), 5 mM MgCl_2_, 50 mM KCl, and 2 mM DTT), 10 mM dNTP each, 20 pmol random hexamer primer and M-MuLV Reverse Transcriptase (Fermentase) in final volume of 25 µl. Then, the suspension was incubated for 1 hour at 42℃. Reverse transcription was terminated by heating at 95℃ for 5 minutes.

OSX expression was analyzed using RT-PCR for cDNA of undifferentiated MSCs and osteoblast differentiated cells in 7^th^, 14^th^ and 21^st^ days of differentiation. Gene expression was normalized using the endogenous GAPDH gene expression as control. For RT-PCR amplification, primer sequences were as follows: OSX forward: GCCAGAAGCTGTGAAACCTC; OSX reveres: GCTGCAAGCTCTCCATAACC; GAPDH forward: CGTCTTCACCACCATGGAGA; GAPDH reverse; CGGCCATCACGCCACAGTTT. These reactions were performed in 2.5 µl 10X PCR buffer (containing: 10 mM Tris-HCl (pH=8.3), 1.5 mM MgCl_2_, and 50 mM KCl), 0.2 mM each dNTP, 0.5 mM each primer, and 1.25 U of Taq DNA polymerase (Fermentase) in final reaction volume of 25 µl. The PCR reaction was done at 95℃ for 5 minutes, then 30 cycles of 94℃ for 30 seconds; 62℃ for 30 seconds; and 72℃ for 30 seconds, followed by one cycle of 72℃ for 10 minutes. The RT-PCR products were analyzed on 1% agarose gel and stained by ethidium bromide.

### Quantitative Real Time-PCR analysis

mRNA expression of candidate gene was quantified by quantitative Real Time-PCR using the LineGene 9600 Florescent quantitative detection system (Hangzhou Bioer Technology, China). Real Time-PCR reactions were prepared by Quantifast SYBR Green PCR kit (Qiagen) according to the manufacturer's instructions. Briefly, reactions were performed in a total volume of 25 µl containing 12.5µl from 2X QuantiFast SYBR Green PCR Master Mix (containing: HotStarTaq Plus DNA Polymerase, QuntiFast SYBR Green PCR Buffer, dNTP mix [dATP, dCTP, dGTP, dTTP], and ROX passive reference dye); 1 µl of cDNA from samples; 0.5µl from each primers; and 10.5µl RNase-Free Water. Real-Time PCR reactions were carried out in triplicate. The real time amplifications included 5 minutes at 95 ℃, followed by 40 cycles at 95 ℃ for 10 seconds and at 60 ℃ for 1 minute. Gene expression levels were normalized to GAPDH expression (ΔCt=Ct_gene of interest_-Ct_GAPDH_), which was used as a housekeeping gene. Results were presented as relative gene expression (2^-ΔCt^).

### Methylation specific PCR

MSP was carried out to determine the methylation status of OSX. MSP was done using specific primers capable of distinguishing between methylated and unmethylated DNA sequences. The MSP primers of OSX were designed by MethPrimer software (27). Primers specific for methylated (M) sequences were as follows: OSXM forward: GTATCGGATAGGCGGAGATC; OSXM reverse: GGTATTGGATAGGTGGAGATTG; and primers specific for unmethylated (U) sequences were as follows: OSXU forward: GGTATTGGATAGGTGGAGATTG; OSXU reverse: AAAACTAATCTAAACAAAACAACAAC. Bisulphite-modified DNA was used for MSP with methylated DNA (treated with Sss1) and normal human unmethylated DNA were used as positive and negative controls, respectively. The PCR mixture contained 10X PCR buffer (500 mM KCl; 100 mM Tris-HCl (pH=8.3); and 15 mM MgCl_2_), dNTPs mix (each at 1.25 mM), primers (0.5 µM each primer), 2 mM MgCl_2_, 1-4% DMSO, 1.25 units of Taq DNA Polymerase (Fermentase), and bisulfite-modified DNA (50 ng) or untreated g DNA (50-100 ng) in a final volume of 25µl. Amplification was carried out in a MyCycler thermal cycler (Bio-Rad) and PCR steps were as follows: 1 cycle of 95℃ for 5 minutes, followed by 35 cycles of 95℃ for 30 seconds; 58℃ for 30 seconds; and 72℃ for 30 seconds, then a final extension at 72℃ for 10 minutes. When using Ferementas Taq DNA polymerase, reactions were manually hot-started at 95℃ for 5 minutes before the addition of 1.25 units of Taq DNA polymerase. Afterwards, the PCR reaction products were separated by electrophoresis in a 1.5% agaros gel and stained with ethidium bromide, and directly visualized under UV illumination.

## Results

### Zoledronic acid induced high extracellular matrix mineralization

**Fig 1:**
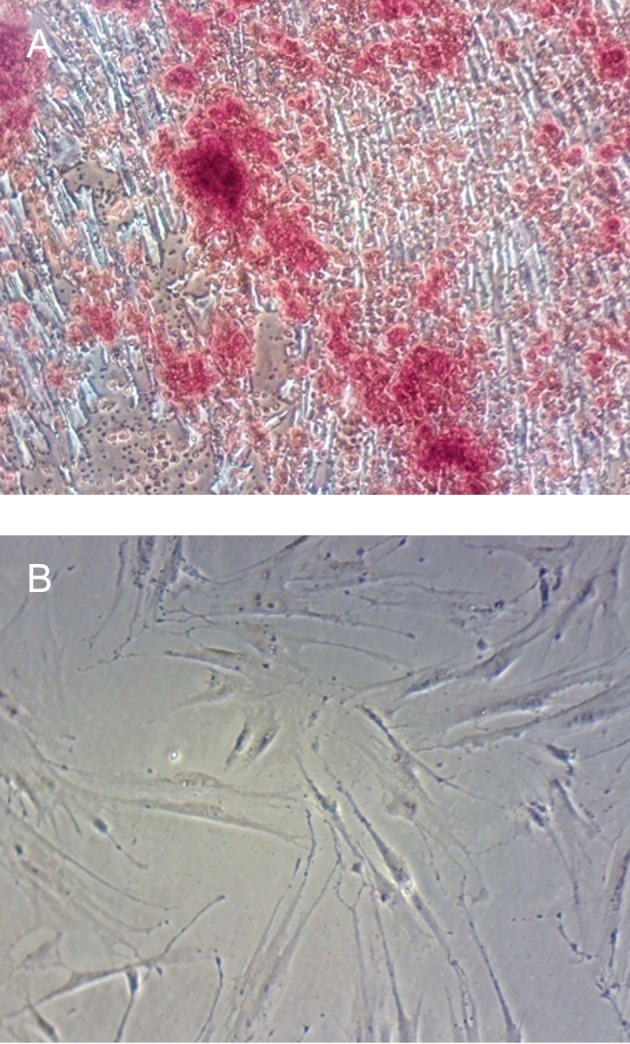
Alizarin Red Staining. A. Zoledronic acid treated MSCs on day 21 and they are positive for Alizarin Red Staining because it causes the calcium deposits turns to red granules. B. Undifferentiated MSCs are negative for Alizarin Red Staining.

Alizarin red staining indicated that the extracellular matrix of MSCs treated with zoledronic acid was uniformly mineralized and significantly greater than the negative control. The MSCs treated with zoledronic acid was stained strongly on day 21 by alizarin red staining, which indicates calcium deposition, whereas undifferentiated MSCs were negative for ARS (Fig 1).

### Qualitative and quantitative analysis of zoledronic acid effect on OSX expression

**Fig 2:**
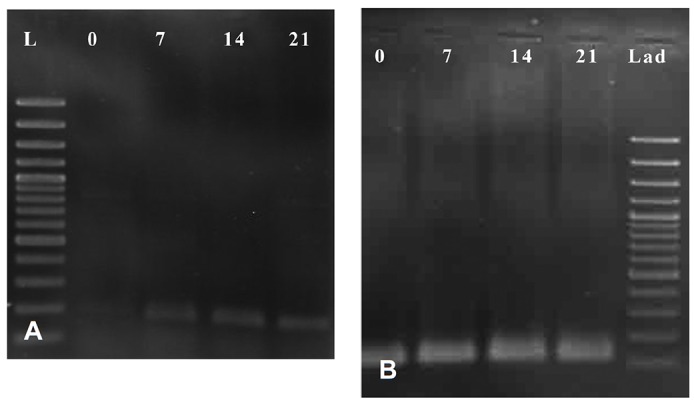
RT-PCR result for OSX(A) and GAPDH(B) expression. RT-PCR for OSX and GAPDH with 161 bp and 130 bp amplicon size, respectively. (0): cDNA of undifferentiated MSC. (7, 14, 21); cDNA of osteoblastic differentiated cells on days 7, 14, and 21, respectively. (L); 100 bp ladder.

**Fig 3:**
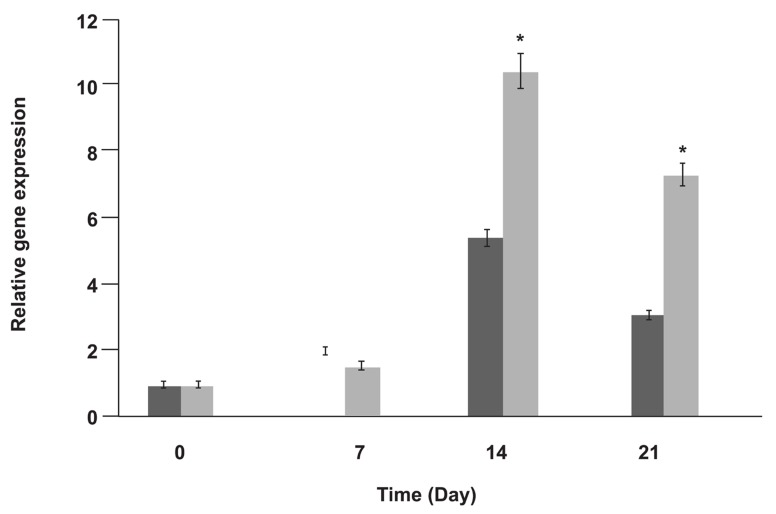
Real Time-PCR for relative gene expression of OSX during osteoblastic differentiation of MSCs with zoledronic acid (grey bars) and without zoledronic acid (black bars), * p<0.05 (comparison of OSX expression with and without zoledronic acid).

To determine the effect of zoledronic acid on OSX expression and evaluate relation between DNA methylation and gene expression, RT-PCR and quantitative real time PCR assays were developed. RT-PCR analysis detected OSX expression in differentiated MSCs during weeks of 1 to 3 of differentiation, but no OSX expression was detected in undifferentiated MSCs (Fig 2). In quantitative Real Time-PCR analysis, OSX expression during differentiation without zoledronic acid showed 2-fold, 5.4-fold and 3.1-fold increase in expression in weeks of 1 to 3 of differentiation, respectively. However, OSX expression during differentiation with zoledronic acid showed 1.6-fold, 10.4-fold and 7.3-fold increase expression in weeks of 1-3 of differentiation, respectively (Fig 3). The mRNA expression of OSX was increased in osteoblast differentiated cells induced by zoledronic acid, especially on days 14 and 21 of differentiation (p<0.05), but expression of OSX didn’t change in undifferentiated MSCs. GAPDH gene expression was used as the control. GAPDH was expressed in both undifferentiated and undifferentiated MSCs during osteoblastic differentiation.

### Effect of zoledronic acid on gene specific methylation of OSX

**Fig 4:**
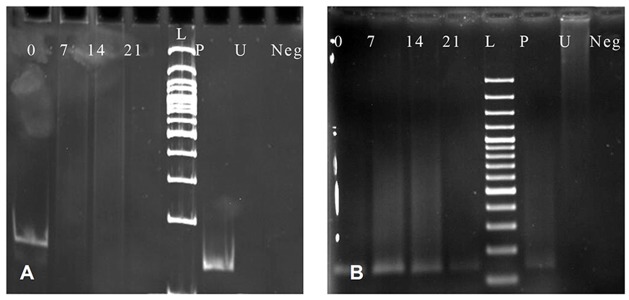
MSP results with M and U primers for OSX. A. MSP with M primers for OSX with 128bp amplicon size. (0); Undifferentiated MSC. (7, 14, 21); osteoblastic differentiated cells on days7, 14, and 21, respectively. (L); 100bp ladder. (P); Positive control as Methylated and SBS treated DNA. (U); SBS Untreated DNA. (Neg); No DNA. B. MSP with U primers for OSX with 130bp amplicon size. (0); Undifferentiated MSC. (7, 14, 21); osteoblastic differentiated cells on days 7, 14, and 21, respectively. (L); 100 bp ladder. (P); Positive control as SBS treated DNA. (U); SBS Untreated DNA. (Neg); No DNA.

To examine the methylation status of the promoter region of OSX in MSCs treated with zoledronic acid, we used Methylation specific PCR. Selected CpG Island in OSX promoter region characterized by UCSC (University of California Santa Cruz) database and span 1146bp. A CpG Island was defined as a DNA sequence with at least 200 bp, and a GC percentage that is greater than 50%, and with an observed-to-expected CpG ratio that is greater than 60% (28). MSP analysis revealed that OSX had a dynamic pattern in methylation while MSCs were gradually differentiated to osteoblasts. On day 0, undifferentiated MSCs were totally methylated. But, on 7 days of differentiation, MSCs treated with zoledronic acid became totally unmethylated. Analysis of OSX promoter afterwards showed a complete unmethylation (Fig 4).

## Discussion

 Osteoporosis is one of the most common health problems in postmenopausal women with great socioeconomic importance (29, 30). Bisphosphonates such as zoledronic acid have major beneficial effects on the skeletal and are one of the most potent class of antiresorptive agents used in the treatment of osteoporosis and other bone disorders (31, 32). These compounds have a high affinity for calcium, so target bone mineral, where they appear to be internalized selectively by bone-resorbing osteoclasts and inhibit their function, promote apoptosis, and reduce bone resorption and bone loss (33). This mechanism in details includes the followings: zoledronic acid inhibits osteoclasts via inhibition of farnesyl diphosphate (FPP) synthase, the cellular biosynthetic, and FPP synthase-mediated mevalonate pathway (4). In the absence of FPP synthase, FPP and geranylgeranyl diphosphate are not produced, which results in the inhibition of the GTP-binding proteins prenylation in osteoclasts. Low levels of prenylated GTP-binding proteins inhibit osteoclast activity and induce osteoclast apoptosis (1, 4, 34).

The majority of *in vitro* studies on zoledronic acid have focused on their action on osteoclastic lineage cells. However, only little attention has been paid to their effects on osteoblastic lineage cells. Recent *in vitro* studies have clearly demonstrated that osteoblastic lineage cells are targets of zoledronic acid, and this compound acts to modulate important cellular function of osteoblasts, including proliferation; differentiation; synthesis of extracellular matrix proteins; and formation of mineralized nodules (7, 35, 36).

In this study, we reported zoledronic acid effect on OSX promoter methylation status during osteoblastic differentiating MSCs treated with zoledronic acid. Our previous studies on osteoblastic differentiation without zoledronic acid treatment revealed that OSX has a dynamic change of methylation pattern while MSCs are gradually differentiated to osteoblasts. Our finding in this research confirm previous findings; whereas, it indicated that zoledronic acid didn’t change methylation pattern of OSX promoter during osteoblastic differentiation of mesenchymal stem cells treated with zoledronic acid. Our findings showed that promoter region of OSX become hypomethylated independent from zoledronic acid treatment during osteoblastic differentiation. This hypomethylation correlates with gene expression, so that OSX expression was observed in osteoblastic differentiated cells; whereas, it is unexpressed in undifferentiated MSCs. Mukherjee et al. showed that expression of OSX in C3H10T1/2 (mouse embryonic fibroblasts) cultured in osteogenic medium with or without BMP2 (bone morphogenetic protein 2) began in days 7 and 5 of differentiation, respectively (37). Another study showed that after 2 weeks of osteogenic differentiation, compared to the control cells, the osteogenic transcription factors OSX and RUNX2 exhibited a more than 2-fold and 5-fold increase in expression, respectively (38). Our study showed that the mRNA expression of OSX increases in osteoblast differentiated cells induced by zoledronic acid, especially in days 14 and 21 of differentiation. This upregulation may be related to other genetic mechanisms. Methyltion pattern of OSX don’t change during zoledronic acid osteoblastic differentiation of MSCs. Our finding showed that zoledronic acid does not induce osteoblastic differentiation via epigenetic mechanisms, especially DNA methylation. However, zoledronic acid can induce osteoblastic differentiation in a manner independent from DNA epigenetic changes. This research focuses on the importance of epigenetic regulation in the osteoblastic differentiation of MSCs treated with zoledronic acid. Finally, we showed that OSX promoter is independently hypomethylated during osteoblastic differentiation of MSCs treated with zoledronic acid.

## Conclusion

In this research, we showed that zoledronic acid does not change methylation pattern of OSX during osteoblastic differentiation of mesenchymal stem cells. This knowledge is important to develop new therapies and treatments to combat disease initiation and progression. However, the interactions between these significant epigenetic marks and the transcriptional downstream regulations needs to be further developed.
